# Multinodal Cervical Angiomyomatous Hamartoma

**DOI:** 10.1177/10668969231212429

**Published:** 2023-11-20

**Authors:** Georgia Mackay, James Johnston, Sameer Mallick, Vinod Khanijow

**Affiliations:** 1 62710 Department of Health Science, The University of Auckland Faculty of Medical and Health Sciences, Auckland, New Zealand; 2 Department of Surgery, 1415 The University of Auckland, Auckland, New Zealand; 3 Department of ORL Head and Neck Surgery, 1387 Auckland District Health Board, Auckland, New Zealand; 4 Department of Pathology, Auckland City Hospital, Auckland, New Zealand

**Keywords:** angiomyomatous hamartoma, cervical lymphadenopathy, lymph node, benign neck mass

## Abstract

Angiomyomatous hamartoma (AMH) is a rare benign lesion of the lymph nodes. Angiomyomatous hamartoma tends to be found in inguinal lymph nodes, and usually in a single lymph node. We present a rare care case of a 53-year-old presenting with a neck lump, found to be AMH involving multiple lymph nodes in her neck. To our knowledge, this is the first case presenting with multiple nodes in this location. There are a limited number of case reports describing magnetic resonance imaging (MRI) features of AMH lesions located in inguinal and head and neck regions. Our MRI findings revealed the mass had intermediate T1 enhancement, high T2 signal enhancement, and high post-gadolinium enhancement and fat saturation of the lesion. Angiomyomatous hamartoma is a histological diagnosis, distinguished from other similar nodal vascular lesions by a number of key features: including the presence of central nodal distribution, muscular blood vessel walls, adipose tissue, and HMB45 negative staining. Early recognition of this benign lesion may have implications for a patient's clinical course and surgical requirements.

## Introduction

An angiomyomatous hamartoma (AMH) is a rare benign lesion of the lymph nodes, there are around 60 reported cases to date.^1,2^ Angiomyomatous hamartoma is characterized histologically by replacement of normal nodal architecture with vascular and smooth muscle elements with or without adipose elements.^1,2^ They tend to present in inguinal and femoral lymph nodes preferentially.^3^ Cases of AMH in the head and neck region are very rare, with less than 10 previous case reports describing AMH in jugular, cervical, submandibular, and post-auricular locations.^3–8^

Angiomyomatous hamartoma usually involves solitary nodes but has been reported to involve multiple nodes in the inguinal regions.^1^ Our literature review has uncovered only 2 previous case reports of AMH involving multiple nodes in inguinal locations and one involving diffuse lymphadenopathy at multiple sites.^1,9,10^

There are 2 cases that describe multiple cervical lymphadenopathy that display histological features of AMH. However, the principal pathology present in both cases was vascular transformation of sinuses, and AMH features were seen in only one or 2 nodes in the specimens.^7,8^ In comparison our case had AMH in all excised nodes. To our knowledge, this is the first case report describing multiple cervical lymphadenopathy with solely AMH features in all involved nodes.

## Case Presentation

Consent for publishing was obtained directly from the patient included in this case report. A 53-year-old female of Samoan ethnicity presented to Otorhinolaryngology clinic, with a 6-month history of a left neck mass that has been slowly enlarging. The mass was 5.5 cm in diameter, and clinically palpable at cervical level V. The mass was painless, without overlying skin changes or fixation to palpable structures. She had no airway compressive symptoms, no dysphagia, odynophagia or dysphonia, and a normal oral and nasopharyngeal examination. Clinically the mass did not appear infectious. She had a 30-pack-year smoking history and a recent intentional 10 kg weight loss.

**Figure 1.**
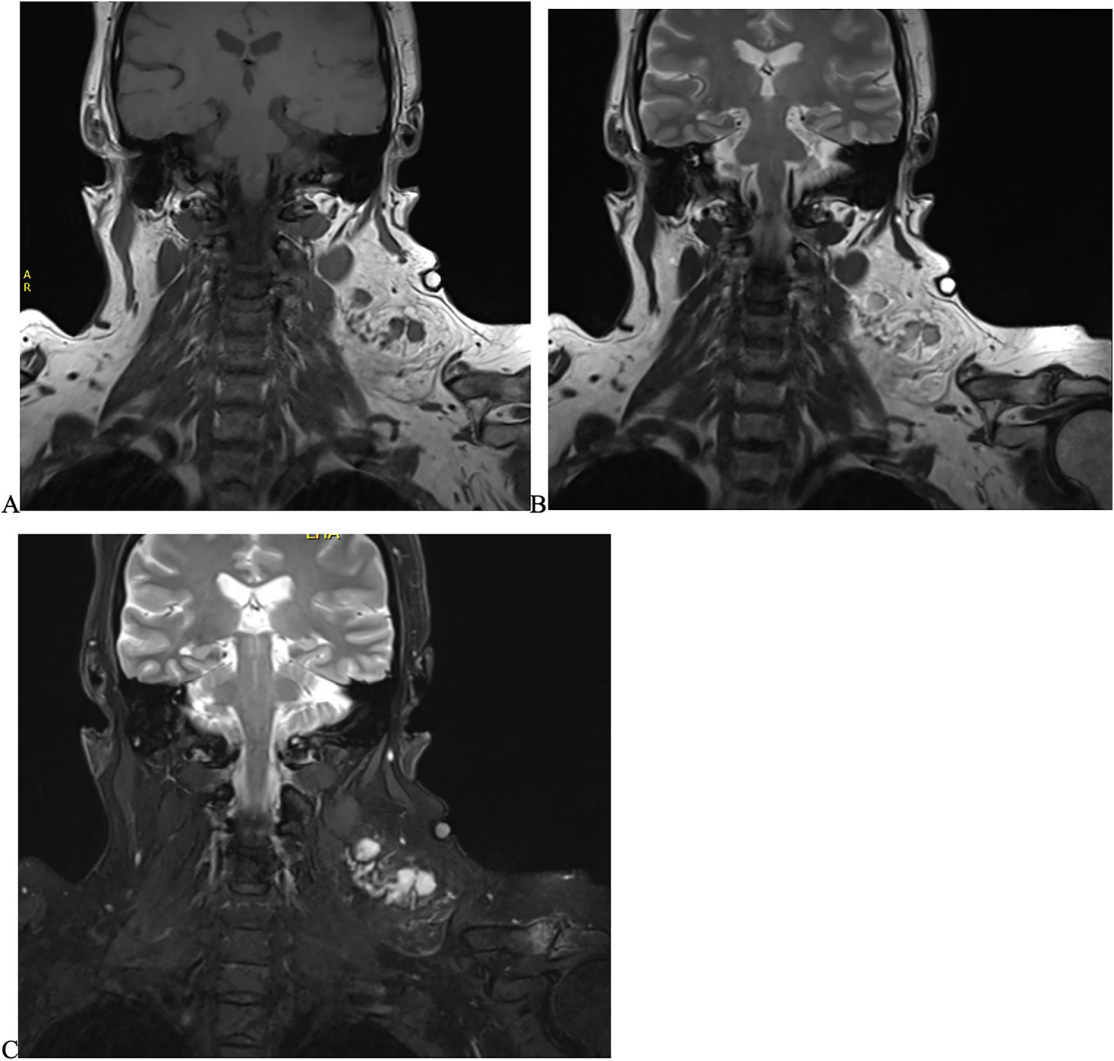
Imaging findings from the patient. Magnetic resonance imaging (MRI) findings show a complex lesion 7×5×6.5 cm in the left posterior triangle of the neck, with solid enhancing components. A, Coronal T1 MRI shows intermediate T1 enhancement of the 6 central solid nodules. (B) Coronal T2 MRI shows high T2 signal enhancement. The lesion does fat saturate. (C) Coronal post-gadolinium (Gd) enhancement shows high Gd enhancement, the nodules have restricted diffusion.

She proceeded for magnetic resonance imaging (MRI) and an FNA (fine needle aspiration). The MRI showed a 7 × 5 × 6.5 cm complex lesion with at least 6 central solid enhancing nodules which had intermediate T1 and high T2 signal and post-gadolinium (Gd) enhancement. Large vessels extended medially from the lesion, while the remainder of the lesion displayed fat saturation consistent with lipomatous lesions. The overall radiological impression was concerning for a liposarcoma (Figure 1).

The FNA showed scant material consistent with a lipomatous lesion, large adipocytes without vascular or stromal elements, and no atypical cells. Following the MRI findings, she underwent a staging CT, revealing no signs of metastatic disease or other unexpected findings. Her case was presented to the Auckland head and neck multidisciplinary meeting to be discussed by experts in pathology, radiology, medical and radiation oncology, and otorhinolaryngology surgery. The consensus was that the radiological features of enhancing solid nodules within a lipomatous lesion were concerning for a liposarcoma. Consequently surgical excision deemed necessary, and at the advise of the multidisciplinary meeting, she underwent a selective left neck dissection of levels 3, 4, and 5b. Intraoperative findings noted significant diffuse adiposity throughout the left neck without discernible tumor capsule.

Macroscopically evaluation of the specimen showed a 78-g fatty mass, measuring 110 × 95 × 60 mm. Upon sectioning, multiple lymph nodes were identified embedded within the adipose tissue.

Microscopically all lymph nodes showed abnormal architecture. The interfollicular region was replaced by collagenous stroma with variably sized vascular channels and a proliferation of spindled cells that in areas, featured fascicular architecture. Admixed intraparenchymal adipose tissue was also noted. Immunohistochemistry with desmin, SMA, SMMHC, and caldesmon highlighted the muscle fibers in the interfollicular region and around these vascular channels of varying caliber, including some abnormally thick proliferations. D2-40 outlined small normal appearing lymphatic channels. HMB45 was negative. The Ki67 proliferation index was <1% in the interfollicular stroma.

**Figure 2.**
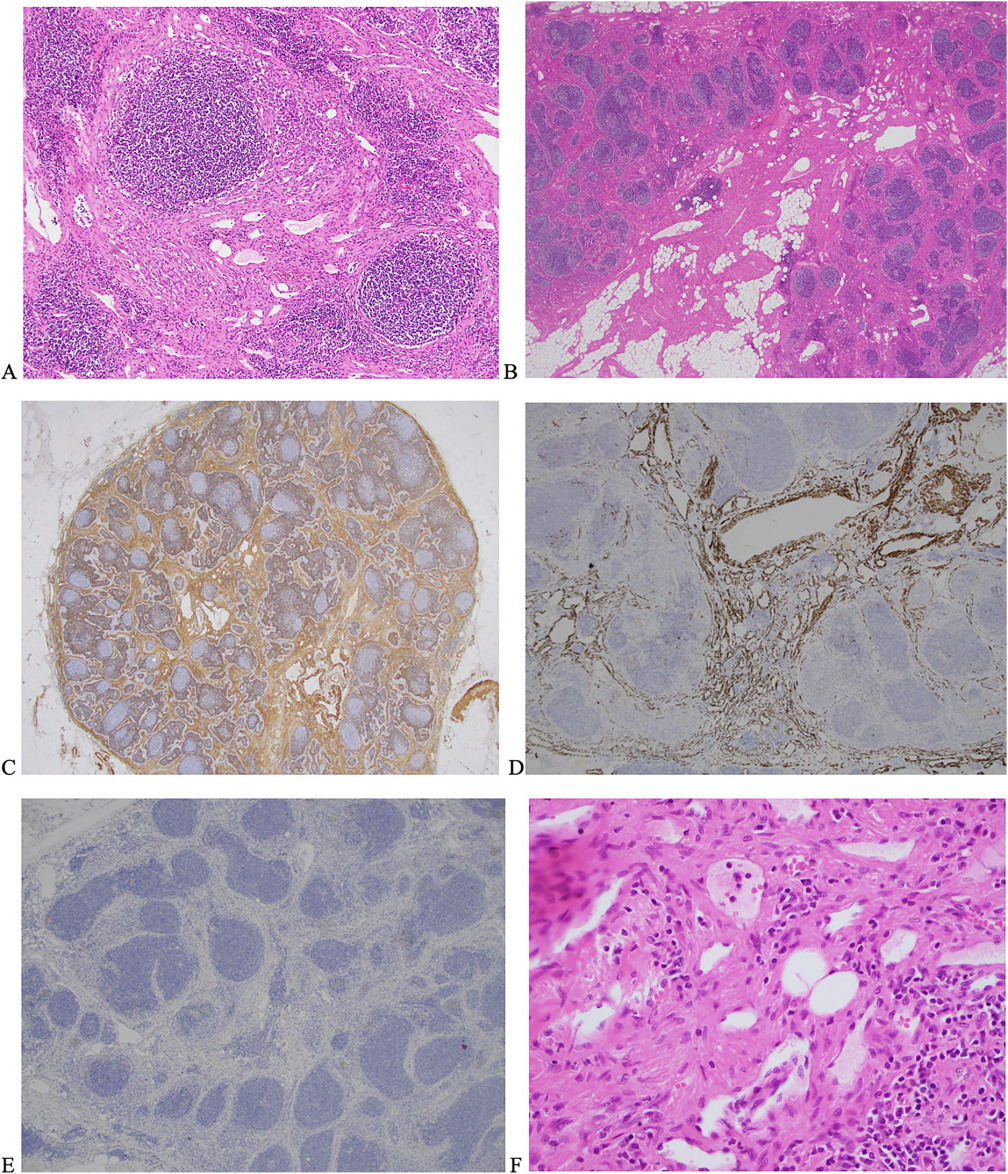
(A and B) Photomicrographs showing in H&E staining of a lymph node within the adipose mass. Original magnification A. ×10 showing dense areas of smooth muscle within sclerotic stroma, characterized by the presence of spindle cells. B (×2 magnification) shows H&E staining with adipose and smooth muscle components replacing the node preferentially at the hilum with extension to the cortex. (C and D) Photomicrographs (C ×2 and D ×4 magnification) which show positive immunohistostaining of smooth muscle fibers in the interfollicular stroma by SMA and desmin staining, respectively. E is a photomicrograph (×4 magnification) showing a negative stain for HMB-45. (F) An H&E staining with magnification ×40 capturing the haphazard arrangement of smooth muscle fibres and fat around small vessels.

There was no cytological atypia, mitosis, or necrosis. The radiological impression of liposarcoma was disproven by the morphologically bland, mature adipose tissue, and conformed with a negative for *MDM2* amplification by fluorescence in-situ hybridization. The overall histology was consistent with AMH of all the resected nodes (Figure 2). The patient progressed well postoperatively, however, developed a seroma after discharge which required readmission and multiple drainages.

## Discussion

Chan et al first described AMH in 1992, as lymph node parenchyma replaced by blood vessels, smooth muscle, and fibrous tissue in the absence of fascicle formation.^11^ Several cases have also noted an adipose component, with some suggesting that the term angiomyolipomatous hamartoma could be used for such lesions.^3^ Clinically and radiologically distinguishing AMH from other soft tissue tumors is difficult due to their similarities in presentation, and hence diagnosis is purely histological.^1^ The differential diagnosis of these lesions prior to biopsy or excision is wide, including reactive lesions, metastatic lesions, sarcomas, and lipoma. Even with histology, diagnosis can be difficult due to other tumor types that have vascular, smooth muscle, and adipose components.^2^ Angiomyomatous hamartoma is differentiated from angiomyolipoma by the lack of prominent perivascular arrangements and a negative stain for HMB-45, such as in our case.^3^ Angiomyolipoma also tends to be present in retroperitoneal nodes and often in the context of renal angiomyolipoma.^12^

Lymphangioleiomyomatosis is a frequent differential diagnosis for AMH lesions due to similarities in their histological characteristics, whereby smooth muscle cells replace normal lymph node parenchyma.^13^ Presenting almost exclusively in middle-aged females lymphangioleiomyomatosis is typically characterized by pulmonary manifestations.^13–15^ Lymphangioleiomyomatosis is a multisystem disorder and frequently involves nodes of multiple sites.^13–15^ In contrast our literature search of AMH, identified only 3 case reports in which AMH involved multiple lymph nodes; 2 of these cases described AMH in inguinal locations, and the third exhibited AMH lymphadenopathy in multiple sites throughout the body.^1,9,10^ Histologically lymphangioleiomyomatosis tends to express compressed irregular lymphatic channels, spindled cells with fascicular pattern and has HMB-45 positive stain.^2,16^ Our case with negative HMB-45, numerous vascular channels, spindled cells without clear fascicular architecture is therefore more suggestive of AMH pathology than of lymphangioleiomyomatosis. Lymphangioleiomyomatosis can also be associated with a genetic disorder, tuberous sclerosis complex, unlike AMH where no such syndromic associations have been reported.^15,16^ Tuberous sclerosis complex is characterized by epilepsy, cognitive impairment, autism, and multiorgan tumor formation.^13–15^ Our case does not have any of these features.^2,16^ The presence of multiple thick-walled vessels within the nodal stroma in this case also contrasts with nodal leiomyomatous lesions which lack the vascular proliferation seen in AMH.^16^ Vascular transformation of sinuses, another nodal vascular lesion, and AMH can be differentiated from each other by a number of distinctive features.^7,8,17,18^ Vascular transformation of sinuses predominantly exhibits capillary blood vessels, which possess minimal muscle wall components, which is in contrast to AMH where well developed muscular blood vessels are present.^17^ Additionally, vascular transformation of sinuses typically initiates its changes in the subcapsular region of the node, while AMH characteristically displays central and hilar region changes in the first instance.^8,17^ Unlike AMH, vascular transformation of sinuses predominately consists of vascular channels and lacks the presence of mature adipose tissue.^17^ In Figure 1, the histological slides clearly demonstrate muscular blood vessels with central nodal distribution and adipose features which are characteristic of AMH.

Most lesions of AMH tend to present with a single node involved.^2^ It is extremely rare to have multiple nodal involvement, to our knowledge to date, there have been only 3 previous cases that have noted multiple site lymphadenopathy.^1,9,10^ Two of which presented in inguinal locations and the third with diffuse lymphadenopathy. We believe this is the first case report of multinodal cervical AMH in the absence of other soft tissue pathology.

Most case reports so far have looked in detail into the histological presentation of this disease, with very few reviewing radiology findings. The cases reporting on MRI findings, like our case, report high T2 signal intensity and post-Gd enhancement.^1^

The pathogenesis of AMH is unknown, some studies hypothesize the impaired lymphatic flow to be a proceeding feature, however, this has not been proven.^1,3^ There have been no reports or recurrences or metastatic disease.^3^ Diagnosis is usually made following excisional biopsy of the involved node; however, there has been one case that has been diagnosed with core biopsy.^16^ As these lesions are benign, conservative management following positive core biopsy can be considered depending on the patients’ surgical risk and symptoms, and in cases of multinodal disease, this could prevent extensive surgical excisions which can pose their own risks to the patient. Angiomyomatous hamartoma, a rare benign vascular pathology, can masqueraded as an aggressive malignant pathology (sarcoma) which resulted in a patient undergoing an invasive surgery. Angiomyomatous hamartoma could be considered in the differential for future similar lesions with lipid and vascular components on imaging, with the option for core biopsy, or excisional biopsy to avoid extensive surgery when doubt on diagnosis is present.

Angiomyomatous hamartoma is a rare benign lymph node pathology which can involve multiple nodes in the head and neck region. While uncommon should be included in the differential for multinodal head and neck lymphadenopathy.
